# Interventions to Reduce Stigma of Dementia: First Insights From a Rural Community-Based Participatory Study

**DOI:** 10.1093/geroni/igaa057.3247

**Published:** 2020-12-16

**Authors:** Juanita Bacsu, Shanthi Johnson, Megan O’Connell, Marc Viger

**Affiliations:** 1 University of Regina, Regina, Saskatchewan, Canada; 2 University of Alberta, Edmonton, Alberta, Canada; 3 University of Saskatchewan, Saskatoon, Saskatchewan, Canada

## Abstract

Age is the greatest risk factor for dementia, and the number of rural older adults is rising. Although dementia-related stigma is widely documented, few studies focus on ways to reduce stigma, especially within rural communities. This late breaker presentation aims to: 1) explore the contributing factors of dementia-related stigma in rural communities; and 2) identify interventions to reduce stigma of dementia in rural communities. Drawing on a community-based participatory approach, data were collected through semi-structured interviews with 18 older adults, and a focus group with 7 community leaders in rural Saskatchewan, Canada. Thematic analysis was used to identify key themes and patterns within the data. Contributing factors of dementia-related stigma ranged from fear to lack of dementia knowledge. Several anti-stigma interventions were identified including: forming support groups; hosting educational workshops; inviting guest speakers with dementia; talking openly about dementia; learning more about dementia; asking questions; sharing your lived-experiences; being inclusive; developing inter-generational programs; and avoiding assumptions and hurtful jokes. As the rural population ages, there is a growing need for interventions, programs, and policies to address stigma of dementia. Engaging in rural partnerships and collaborative research is essential to developing community-informed strategies to reduce dementia-related stigma and improve the quality of life for people with dementia.

